# Therapeutic effects of forest bathing on older adult patients with essential hypertension: evidence from a subtropical evergreen broad-leaved forest

**DOI:** 10.3389/fpubh.2025.1631613

**Published:** 2025-07-30

**Authors:** Aibo Li, Yu Liu, Haiyuan Qian, Kun Sun, Ziqing Zhao, Iwao Uehara, Guofu Wang, Benzhi Zhou

**Affiliations:** ^1^Research Institute of Subtropical Forestry, Chinese Academy of Forestry, Hangzhou, Zhejiang, China; ^2^Qianjiangyuan Forest Ecosystem Research Station, National Forestry and Grassland Administration, Zhejiang, Hangzhou, China; ^3^Qianjiangyuan National Park Administration Bureau, Quzhou, Zhejiang, China; ^4^Faculty of Regional Environment Science, Tokyo University of Agriculture, Tokyo, Japan; ^5^Department of Geriatrics, Zhejiang Hospital and Zhejiang Provincial Key Laboratory of Geriatrics & Geriatrics Institute of Zhejiang Province, Zhejiang, Hangzhou, China

**Keywords:** forest bathing, older adult patients with essential hypertension, subtropical evergreen broad-leaved forest, blood pressure regulation, heart rate variability, psychological well-being

## Abstract

**Introduction:**

Forest bathing (Shinrin-Yoku) has gained growing attention in medical and therapeutic tourism research due to its potential benefits in managing chronic diseases, such as hypertension. This empirical study examined the therapeutic effects of forest bathing on older adult patients with essential hypertension.

**Methods:**

A total of 36 participants were randomly assigned to either a forest environment (experimental group, *n* = 24) or an urban setting (control group, *n* = 12) for a three-day, two-night intervention. To minimize potential confounding factors, both groups followed identical dietary regimens, leisure activities, and sleep schedules throughout the intervention. Physiological and psychological assessments, including vital signs, inflammatory markers, heart rate variability (HRV), and mood states, were conducted at baseline and post-intervention.

**Results:**

The results indicated that systolic blood pressure (SBP), diastolic blood pressure (DBP), and high-sensitivity C-reactive protein (hs-CRP) levels were significantly lower in the experimental group compared to the control group (*p* < 0.05). Frequency domain parameters of HRV, specifically LF and the LF/HF ratio, significantly increased in the experimental group (*p* < 0.05). Additionally, psychological assessments revealed that participants exposed to the experimental group had significantly better emotional well-being. Specifically, tension-anxiety scores decreased significantly, while vigor-activity scores increased (*p* < 0.05).

**Discussion:**

These findings suggest that forest bathing can serve as an effective non-pharmacological intervention for reducing blood pressure, improving autonomic function and mental health among older adult patients with essential hypertension. This study provides empirical evidence supporting the therapeutic potential of forest environments, particularly subtropical broad-leaved evergreen forests, in the integrated management of cardiovascular and mental health.

## 1 Introduction

Forests, as a crucial component of terrestrial ecosystems, offer aesthetic landscapes, favorable microclimatic conditions, and rejuvenating atmospheric quality, making them ideal settings for human leisure and wellness activities (1–3). Notably, forests emit substantial amounts of negative air ions and various bioactive volatile organic compounds, both of which have been extensively documented for their therapeutic effects on human health (4, 5). Forest environments have been shown to modulate the autonomic nervous system (ANS), enhance endocrine homeostasis, and exhibit anti-inflammatory and antioxidant effects, thereby contributing to overall cardiovascular health (2, 6). These mechanisms provide a solid basis for exploring forest-based interventions for chronic diseases, particularly hypertension (7, 8).

Forest bathing (Shinrin-Yoku), originally developed in Japan in the 1980s, refers to the practice of immersing oneself in a forest environment using all five senses to promote relaxation and health. It involves slow, mindful walks, deep breathing, and sensory engagement with natural elements, without the need for physical exertion or specific exercise routines (1, 9). The increasing popularity of forest bathing and forest therapy has led to a growing recognition of their physical and psychological healing effects (10). Since the Forest Therapy Index was released in 2017, China has systematically developed its forest healthcare system by integrating forestry, medical care, older adult care, and tourism, reflecting a growing institutional effort to harness natural environments for public health (11). Previous studies have demonstrated that forest exposure may regulate autonomic nervous system activity, enhance immune responses, and reduce psychological stress (5, 12, 13). Increasingly, such interventions are conceptualized globally under the framework of nature-based interventions (NBI), including green prescribing (GRx) and nature-based prescribing (NBP). These approaches integrate nature exposure into clinical and public health programs as evidence-based strategies for managing chronic conditions. For instance, a comprehensive systematic review by Nguyen et al. (14) reported that nature prescriptions significantly reduced both systolic (−4.82 mm Hg) and diastolic (−3.82 mm Hg) blood pressure. These findings underscore the relevance of positioning forest bathing within a broader evidence-based preventive health paradigm, especially for managing chronic conditions such as hypertension in aging populations (6, 8).

Hypertension is a chronic systemic condition characterized by persistently elevated arterial pressure, potentially causing complications in the cardiovascular system, brain, and kidneys (15). Individuals aged 65 years or older are at the highest risk, and hypertension is clinically defined by sustained systolic blood pressure ≥140 mmHg or diastolic blood pressure ≥90 mmHg (16–18). According to reports, essential hypertension accounts for over 95% of hypertension cases, referring to elevated blood pressure without a clear cause, which may result from the combined effects of genetic and environmental factors (18–20). Given the limitations of pharmacological treatments, including concerns about side effects, medication adherence, and long-term dependency, non-pharmacological interventions such as lifestyle modifications, physical activity, and environmental therapies have gained increasing attention (21, 22).

Among various natural environments, subtropical evergreen broad-leaved forests stand out due to their stable climate, abundant bioactive volatile organic compounds, and high negative air ion concentrations, which offer optimal conditions for cardiovascular health restoration (23). Although previous studies have reported the blood pressure-lowering effects of forest bathing, empirical evidence regarding its therapeutic efficacy in older adult patients with essential hypertension remains limited (8, 25). This gap is particularly evident in subtropical forest ecosystems, which, despite their ecological, economic, and recreational significance, remain understudied in the context of hypertension management through forest therapy (8, 11). To address this knowledge gap, our study conducted activities such as forest walking, meditation, tea ceremony performances, and eight trigrams boxing exercises in a forest environment, with the aim of evaluating the adjunctive therapeutic potential of forest bathing for older adult patients with essential hypertension in a subtropical evergreen broad-leaved forest setting. The findings will not only advance the scientific understanding of forest-based health interventions but also provide empirical support for integrating forestry and healthcare sectors, potentially contributing to evidence-based forest therapy programs and public health policies.

## 2 Materials and methods

### 2.1 Experimental sites

**Figure 1:**
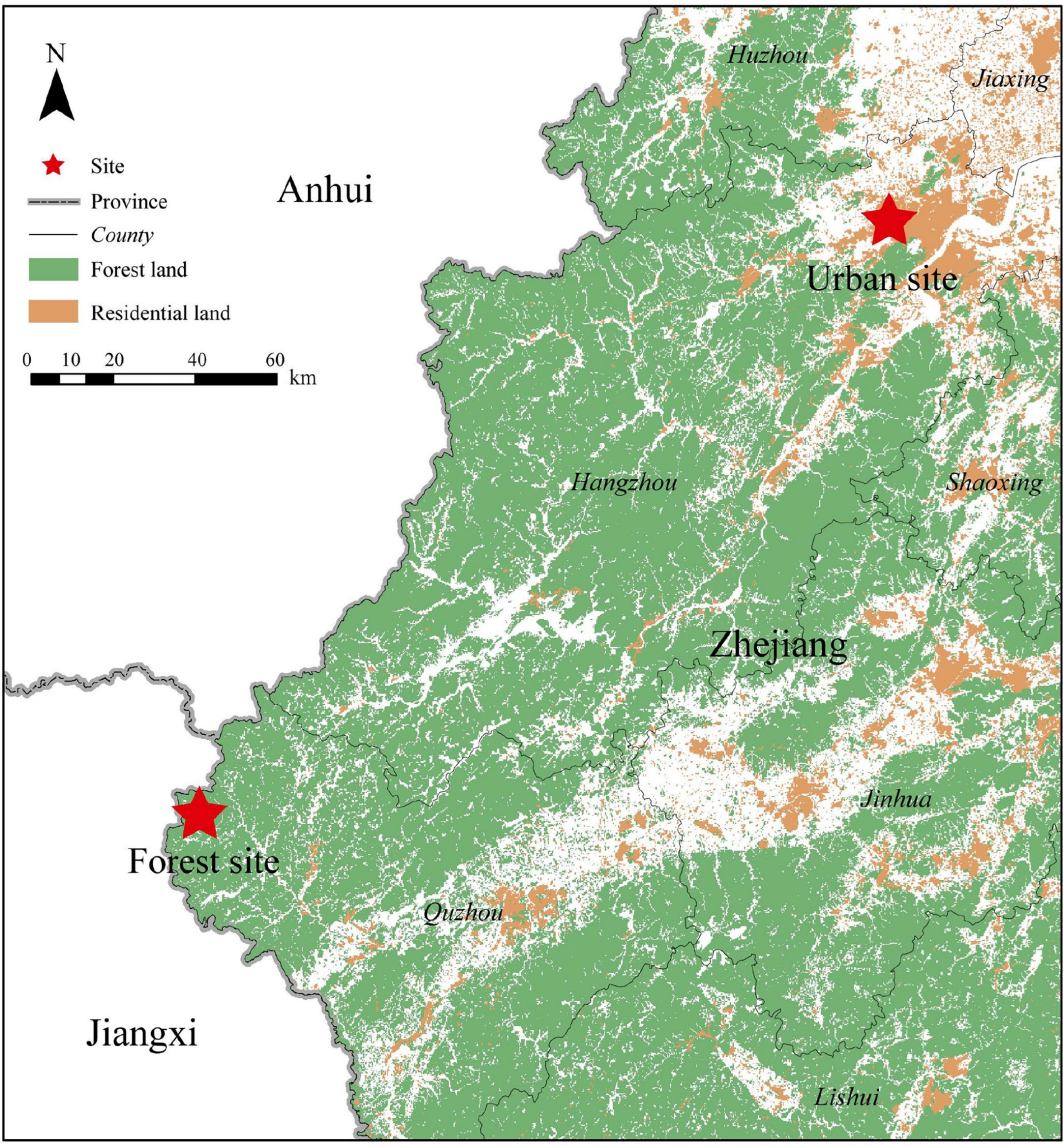
Location of the experimental sites.

This experiment was conducted at two distinct sites: an urban environment in Hangzhou City and a forest environment in Qianjiangyuan National Park, located in Kaihua County, Zhejiang Province, China (Figure 1). It has a subtropical monsoon climate with four distinct seasons, a mean annual temperature of 16.4°C, average annual precipitation of 1814 mm, 1712.5 sunshine hours, and a 252-day frost-free period.

The urban site, located in the bustling commercial district of Hangzhou City (120°5′49″E, 30°18′6″N), represents a typical high-density metropolitan environment in eastern China. Hangzhou is the capital of Zhejiang Province and a major economic and cultural center with a population exceeding 12 million. The study site is characterized by high-rise buildings, heavy vehicular traffic, high ambient noise levels, limited greenery, and frequent air pollution episodes, making it representative of the daily living environment for most urban residents.

The forest site is located in Qianjiangyuan National Park (118°7′0″E, 29°14′26″N), approximately 220 km away from the Hangzhou experimental site as the crow flies. The travel time by car is about 3.5 h under normal road conditions. The park covers 252 km^2^ and is located in the mountainous region of western Zhejiang. It is primarily composed of subtropical evergreen broad-leaved forests, including species such as *Castanopsis eyrei*, *Lithocarpus glaber*, *Cyclobalanopsis glauca*, and *Schima superba*. Other vegetation types include evergreen-deciduous broadleaved mixed forests, coniferous-broadleaved mixed forests, coniferous forests, and subalpine wetlands. The park is recognized as a natural gene pool in eastern China, harboring 184 families, 827 genera, and 1852 species of vascular plants. Among them, 805 species have ornamental value, 770 are medicinal plants, 122 are edible, 266 produce aromatic oils, and 110 are fiber plants. The faunal diversity includes 104 bird species, 58 mammals, 26 amphibians, 51 reptiles, and 1,156 insect species, supporting the park’s designation as an ecologically significant area.

In terms of infrastructure, the park is well-equipped to support nature-based tourism and scientific research, featuring paved forest trails, visitor centers, scenic pavilions, and ecological parking areas. The forest landscape offers diverse sensory stimuli, including the scent of aromatic plants, the sound of flowing streams and bird calls, seasonal wildflowers, dense green canopies, and sweeping mountain vistas, which together create an immersive, tranquil natural environment conducive to forest bathing and ecological studies. By contrast, the urban environment lacks such restorative features and is characterized by sensory overstimulation, including traffic noise, artificial lighting, heat-retaining surfaces, and limited green space. The stark contrast between the two sites provided a suitable basis for investigating the physiological and psychological effects of forest bathing on hypertensive older adult patients.

### 2.2 Subjects and study design

#### 2.2.1 Subjects

Participants were recruited from Hangzhou City prior to the experiment. Thirty-six eligible participants were selected based on these inclusion criteria: (1) diagnosed with essential hypertension; (2) aged between 60 and 80 years (born May 21, 1941, to May 20, 1961); (3) Class I-II cardiac function according to the New York Heart Association (NYHA) classification; (4) capable of self-care and independent daily activities. Exclusion criteria included: (1) cognitive impairments affecting communication, such as severe vision or hearing disorders; (2) severe chronic illnesses, including cancer or serious cardiac, pulmonary, hepatic, renal, or central nervous system conditions; (3) a history of acute myocardial infarction within the last 3 months, stroke within the last 6 months, or recent major surgery or severe trauma; (4) acute infections (e.g., respiratory or gastrointestinal) within 2 weeks before or during the trial period; (5) uncontrolled hypertension with systolic blood pressure ≥180 mmHg and/or diastolic blood pressure ≥110 mmHg despite medication.

All participants were community-dwelling individuals who received occasional outpatient care and were not hospitalized patients. Although no formal psychiatric diagnoses were assessed, individuals with known mental disorders or emotional instability were excluded. Baseline Profile of Mood States (POMS) scores further indicated that participants did not exhibit severe psychological distress.

All procedures were approved by the Ethics Committee of Zhejiang Hospital (Grant No. 2021, Preliminary Review No. 13 K) and complied with the Declaration of Helsinki (1975, revised 1983). Written informed consent was obtained from all participants after being fully informed of the study procedures and potential adverse events. Possible adverse events included transient dizziness, fatigue, mild headache, or elevated blood pressure related to environmental or physical changes; however, no serious adverse events occurred during the study.

#### 2.2.2 Study design

Participants were randomly allocated to two groups at a 1:2 ratio using a computer-generated randomization method, resulting in a control group (*n* = 12) and an experimental group (*n* = 24). The study period was from May 21 to May 23, 2021. Participants received a briefing on the experimental protocol on the evening of May 20. On the morning of May 21, prior to breakfast, baseline blood samples were collected at the Sandun Branch of Zhejiang Hospital. Subsequently, participants were transported to their assigned intervention sites: the experimental group was taken to a forest environment in Qianjiangyuan National Park, while the control group remained in an urban setting representative of a typical busy city district.

The forest bathing intervention was conducted in a subtropical evergreen broad-leaved forest and included a series of structured, low-intensity activities designed to enhance sensory engagement with the natural environment. These included: (1) eight trigrams boxing—a traditional Chinese Qigong practice involving slow, coordinated movements and deep breathing, performed for 1 h daily; (2) forest walking—gentle hiking along designated forest trails for approximately 3 h daily, interspersed with rest periods to encourage mindful observation of the surrounding sights and sounds; (3) tea ceremony—a one-hour traditional tea ceremony performance held in the park’s activity room on the afternoon of May 21 to promote relaxation and social interaction; and (4) meditation—a one-hour mindfulness meditation session conducted under a forest pavilion on the afternoon of May 22.

**Figure 2:**
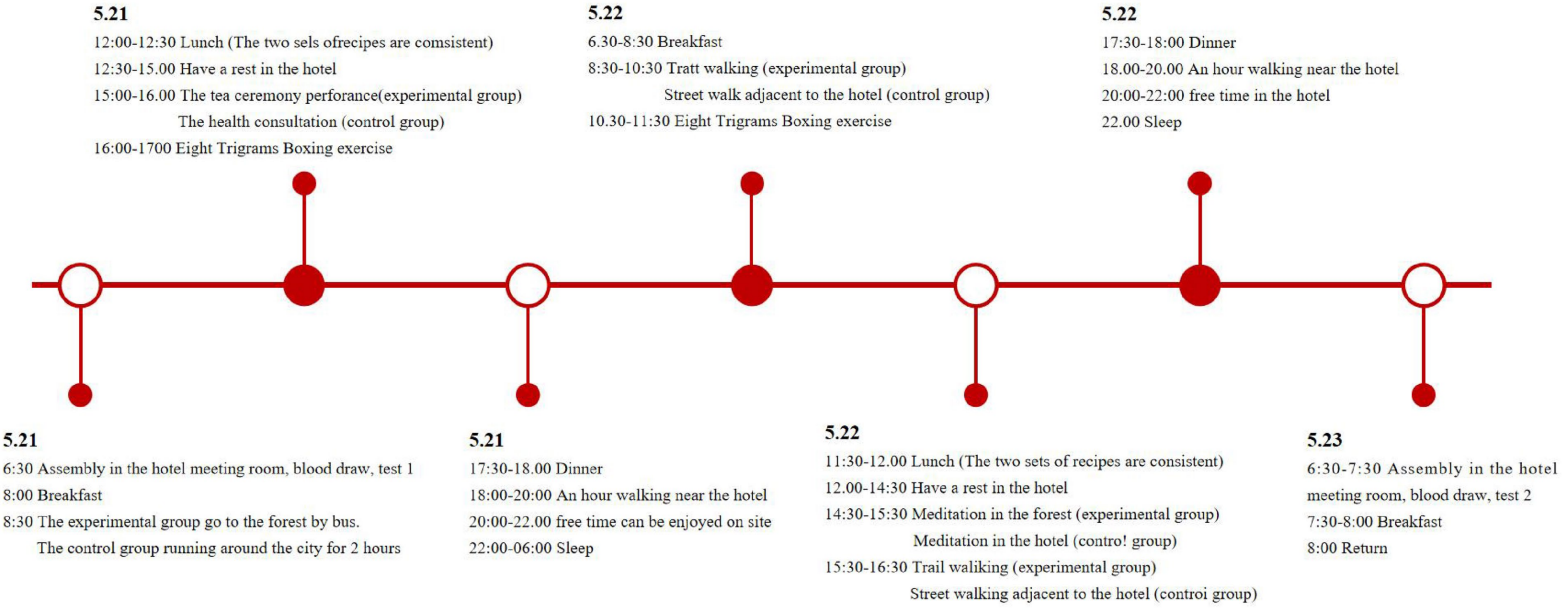
Activity schedule of experiment.

**Figure 3:**
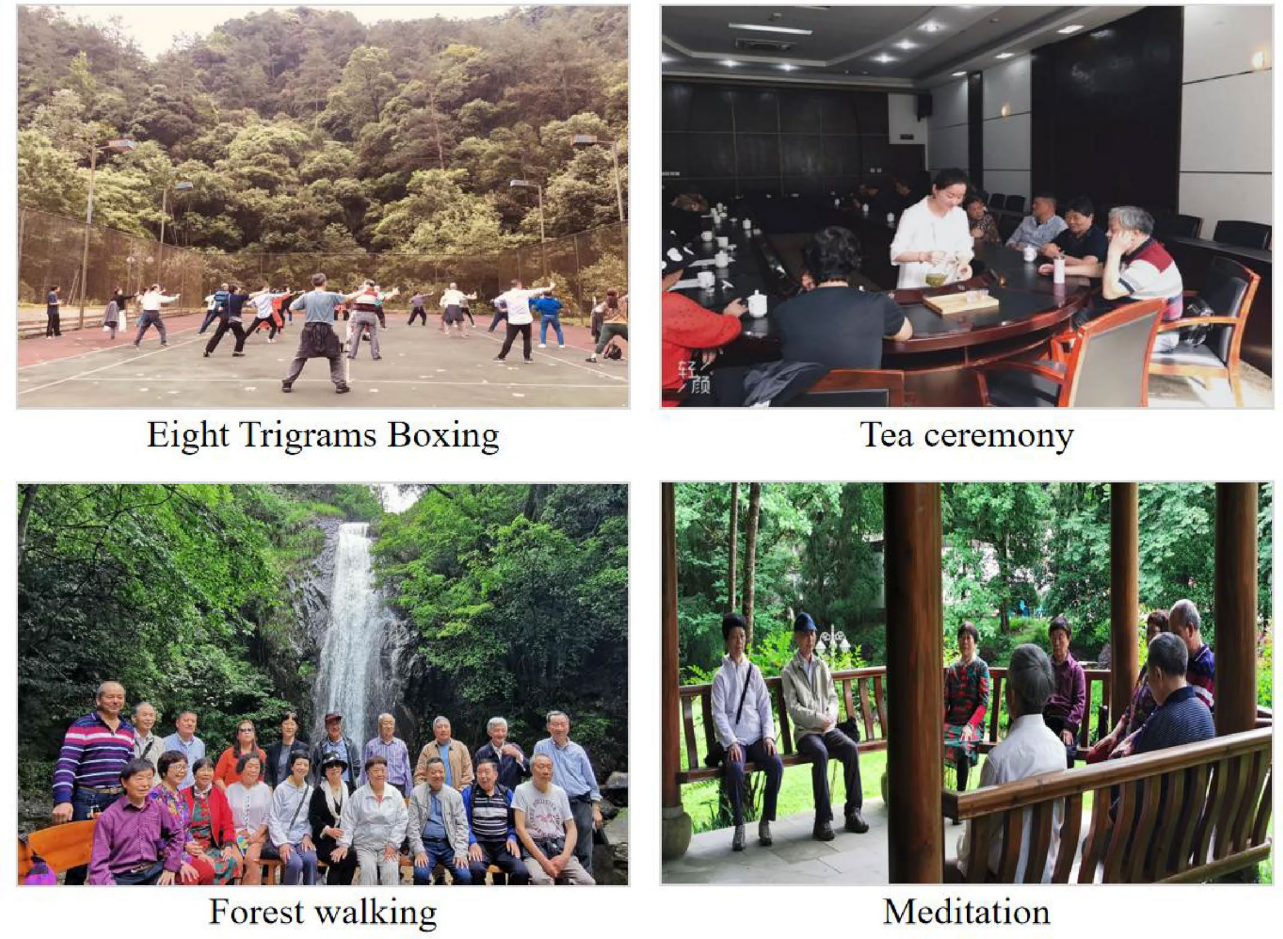
Activity items of experiment.

Both groups followed identical schedules for diet, leisure activities, and sleep to minimize potential confounding variables. Participants were restricted from using electronic devices and engaging in physical exertion, tobacco, alcohol, and caffeine consumption during the study. The detailed experimental schedule is provided in Figures 2, 3.

#### 2.2.3 Blood pressure, heart rate, oxygen saturation, and heart rate variability measurements

Blood pressure (BP) and heart rate (HR) were measured using a portable digital sphygmomanometer (Omron HEM-7000-E, Kyoto, Japan). Oxygen saturation (SpO_2_%) was assessed using a portable pulse oximeter (YUWELL YX301, China). Heart rate variability (HRV), including low frequency (LF) power, high frequency (HF) power, and the LF/HF ratio, was measured using a Bluetooth-enabled HRV sensor (Fujian Heart Biotechnology Co., Ltd., Fujian, China). All physiological parameters were measured between 6:30 am and 7:30 am, before and after the intervention, ensuring consistency and minimizing potential diurnal variation.

#### 2.2.4 Inflammatory markers measurements

To evaluate inflammatory responses, high-sensitivity C-reactive protein (hs-CRP) and interleukin-6 (IL-6) were measured using ELISA-based assays (Nanjing Jiancheng Bioengineering Institute, Nanjing). Oxidative stress markers, including glutathione peroxidase (GSH-Px; Beyotime Biotechnology, Shanghai) and malondialdehyde (MDA; Elabscience Biotechnology, Wuhan), were also quantified. Additionally, plasma cortisol levels were assessed as an indicator of hormonal stress responses using ELISA kits (Elabscience Biotechnology, Wuhan). Blood samples for these analyses were collected consistently between 6:30 am and 7:30 am, both before and after the intervention.

#### 2.2.5 Emotional state assessment

The POMS questionnaire was used to assess mood changes. This 65-item self-administered scale evaluates six dimensions: tension-anxiety (T), depression-dejection (D), fatigue-inertia (F), confusion-bewilderment (C), vigor-activity (V), and anger-hostility (A). Mood state assessments were conducted both pre-and post-intervention, with data collection occurring between 6:30 am and 7:30 am. The post-intervention assessment was carried out at the hotel where participants were accommodated. On average, participants completed the questionnaire within approximately 10 min. This standardized timing and controlled indoor environment were designed to minimize variability and ensure consistency across measurements.

### 2.3 Data analysis

Statistical analysis was performed using SPSS (version 24.0) and GraphPad Prism (version 7.0). Normality of data distribution was assessed using the Shapiro–Wilk test, and homogeneity of variance was evaluated using Levene’s test (26, 27). Different statistical methods were employed depending on the distribution characteristics and variance homogeneity of the data. An independent t-test was used to compare groups when data exhibited normal distribution and homogeneous variance. The Mann–Whitney U test was applied for non-normally distributed independent samples, whereas the Wilcoxon signed-rank test was used for paired samples (28, 29). Furthermore, Pearson’s correlation analysis was conducted to examine relationships between mood states and physiological parameters. Statistical significance was set at *p* < 0.05 for all analyses.

## 3 Results

### 3.1 Baseline clinical data for the subjects

At baseline, no significant differences in age, systolic blood pressure (SBP), diastolic blood pressure (DBP), HR, SpO_2_%, HRV, inflammatory markers, or mood states were observed between the experimental and control groups (Table 1). All *p*-values exceeded 0.05, indicating comparable baseline characteristics between the two groups. This statistical equivalence ensured that any subsequent differences observed could be attributed to the intervention rather than pre-existing disparities between the groups.

**Table 1:** Baseline level of the indicators of subjects before the experiment.

Indicators		Control group (*n* = 12)	Experimental group (*n* = 24)
Gender (male/female)		7/5	13/11
Age (year)		73.75 ± 6.41	71.92 ± 6.06
Systolic blood pressure (mmHg)		148.58 ± 19.07	140.79 ± 18.35
Diastolic blood pressure (mmHg)		85.67 ± 14.45	81.46 ± 9.06
Heart rate (bpm)		72.42 ± 11.57	76.5 ± 11.38
Oxygen saturation (%)		97.92 ± 1.38	98.29 ± 1.16
Heart rate variability	LF (m/s^2^)	180.44 ± 31.86	165.03 ± 56.7
	HF (m/s^2^)	88.48 ± 54.8	77.7 ± 23.31
	LF/HF	2.79 ± 1.45	2.17 ± 0.6
	hs-CRP (ng/l)	59.99 ± 18.34	65.36 ± 50.545
	IL-6 (ng/l)	749.61 ± 215.52	767.06 ± 340.26
	Cortisol (ug/l)	250.58 ± 166.56	336.72 ± 618.01
	MDA (ug/l)	293.06 ± 87	263.88 ± 69.26
	GSH-Px (units)	14.62 ± 18.48	9.97 ± 3.92
Profile of mood states	Tension-anxiety	13.25 ± 3.39	12.75 ± 2.88
	Depression-dejection	22.92 ± 5.74	19.58 ± 5.05
	Anger-hostility	19.08 ± 3.42	17.92 ± 2.80
	Vigor-activity	23.42 ± 3.6	25.33 ± 6.93
	Fatigue-inertia	12.67 ± 2.39	11.54 ± 2.02
	Confusion-bewilderment	13.67 ± 2.87	13.33 ± 2.67

### 3.2 Effect of forest bathing on BP, HR, and SpO_2_%

**Figure 4:**
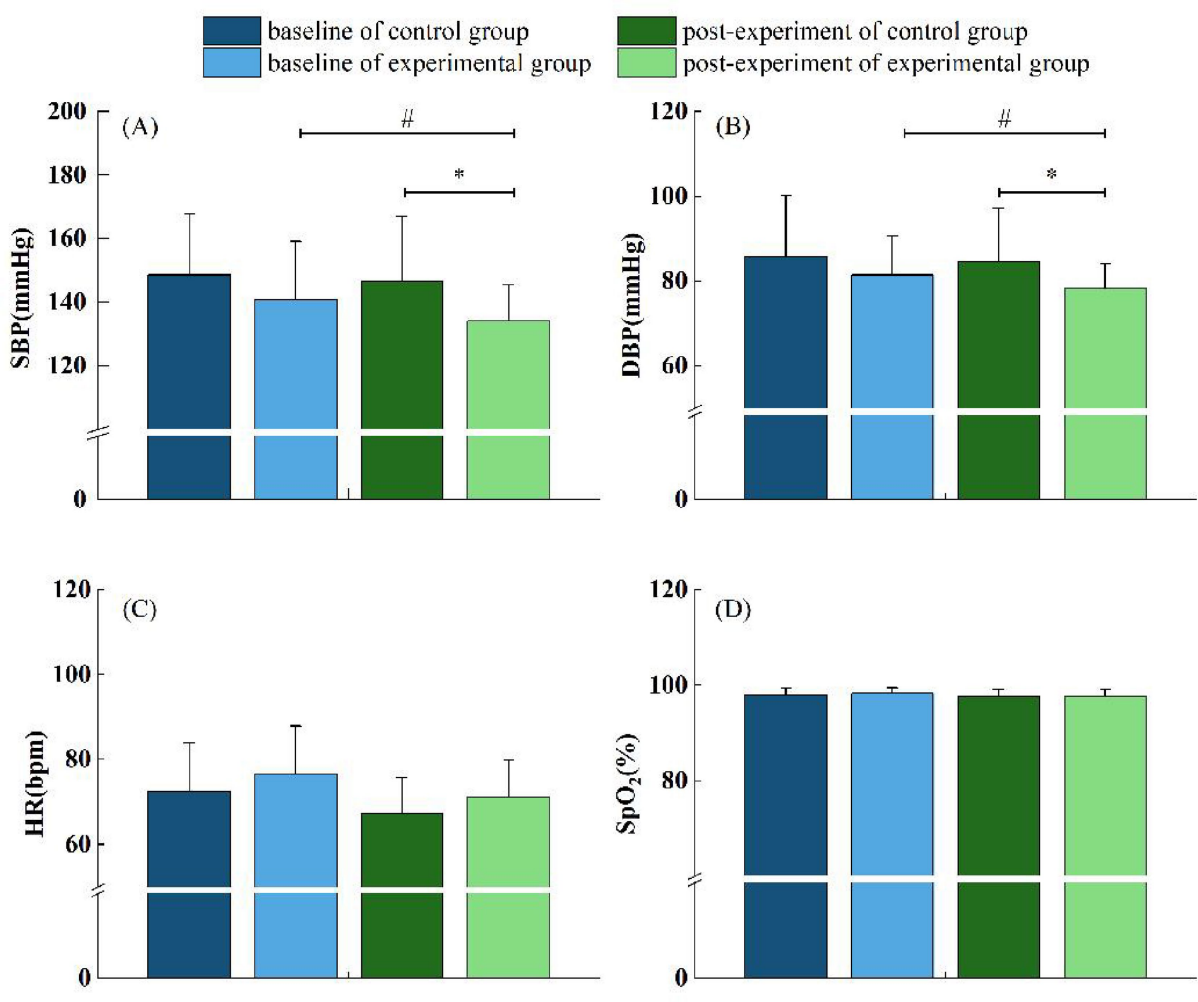
**(A–D)** Effects of forest bathing on BP, HR, and SpO_2_% between the control and experimental groups. *n* = 12 (in control) and *n* = 24 (in experimental). *p < 0.05 analyzed with independent sample *t*-test. ^#^*p* < 0.05 analyzed with paired sample *t*-test. The same figure below.

After the intervention, significant reductions in SBP and DBP were observed in the experimental group compared to the control group. SBP in the experimental group decreased significantly to 134.08 ± 11.33 mmHg, lower than that in the control group (146.50 ± 20.62 mmHg, *p* < 0.05). Similarly, DBP was significantly lower in the experimental group (78.25 ± 5.71 mmHg) than in the control group (84.58 ± 12.61 mmHg, *p* < 0.05). In contrast, no statistically significant differences were detected between the groups regarding HR (71.25 ± 8.5 bpm vs. 67.25 ± 8.42 bpm, *p* > 0.05) or SpO_2_% (97.75 ± 1.29% vs. 97.67 ± 1.5%, *p* > 0.05) (Figure 4). These results suggest that forest bathing could effectively lower blood pressure among older adult hypertensive individuals, however, its effects on HR and oxygen saturation were not evident within the limited duration of this study.

### 3.3 Effect of forest bathing on HRV

**Figure 5:**
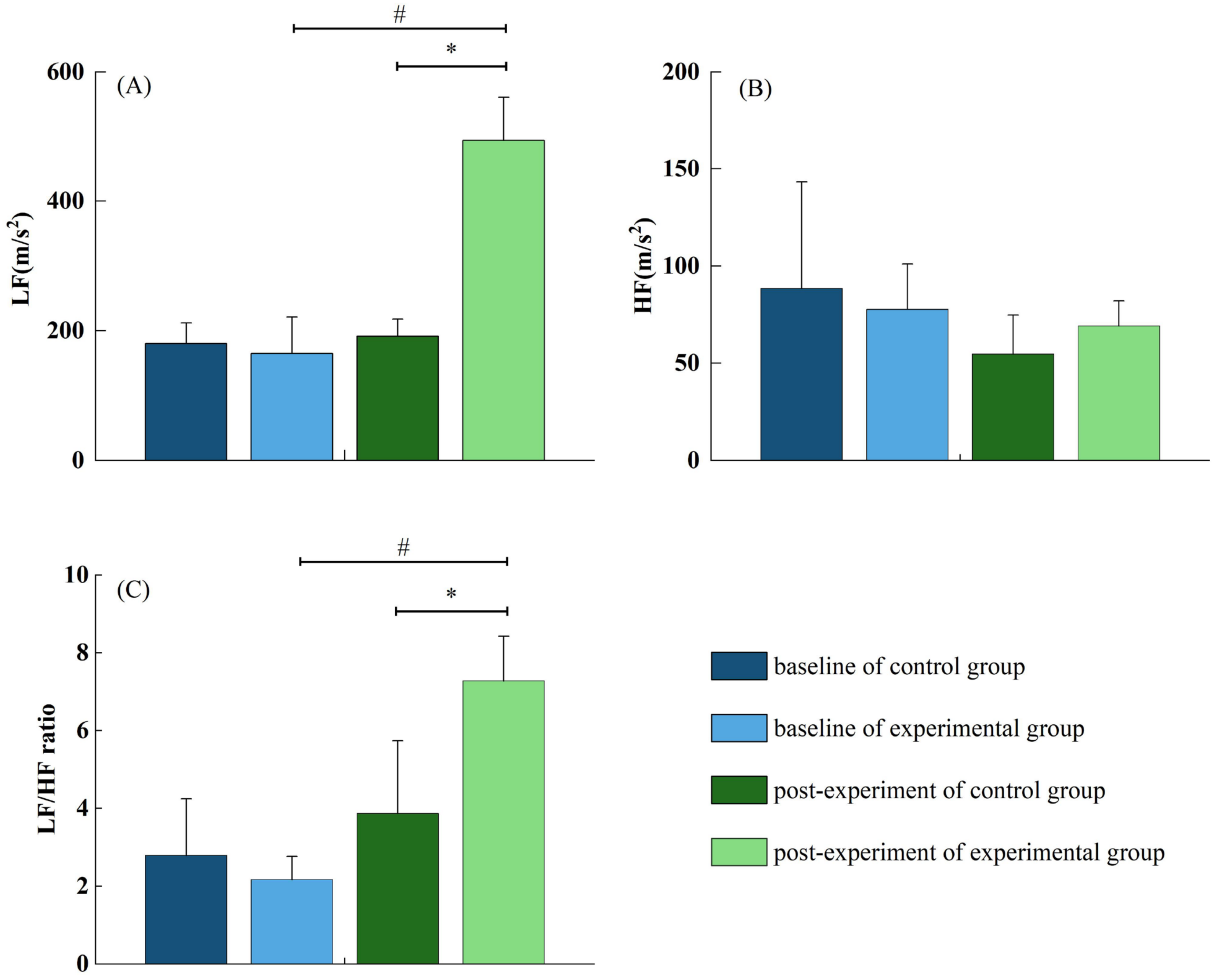
**(A–C)** Effects of forest bathing on LF, HF, and LF/HF between the control and experimental group.

Heart rate variability, a well-established indicator of autonomic nervous system function and cardiovascular regulation, demonstrated significant alterations in response to forest bathing. Compared with the control group, the experimental group exhibited a substantial increase in LF power (191.54 ± 26.67 vs. 494.22 ± 66.87, *p* < 0.05) and in the LF/HF ratio (3.87 ± 1.87 vs. 7.28 ± 1.15, *p* < 0.05), suggesting a shift toward enhanced sympathetic modulation. However, the difference in HF power between the experimental and control groups was not statistically significant (60.54 ± 14.26 vs. 48.37 ± 14.42, *p* > 0.05) (Figure 5). These results imply that forest bathing may modulate autonomic nervous system activity by increasing sympathetic nervous system dominance, potentially contributing to cardiovascular homeostasis and stress adaptation in older adult patients with essential hypertension.

### 3.4 Effect of forest bathing on inflammatory markers

**Figure 6:**
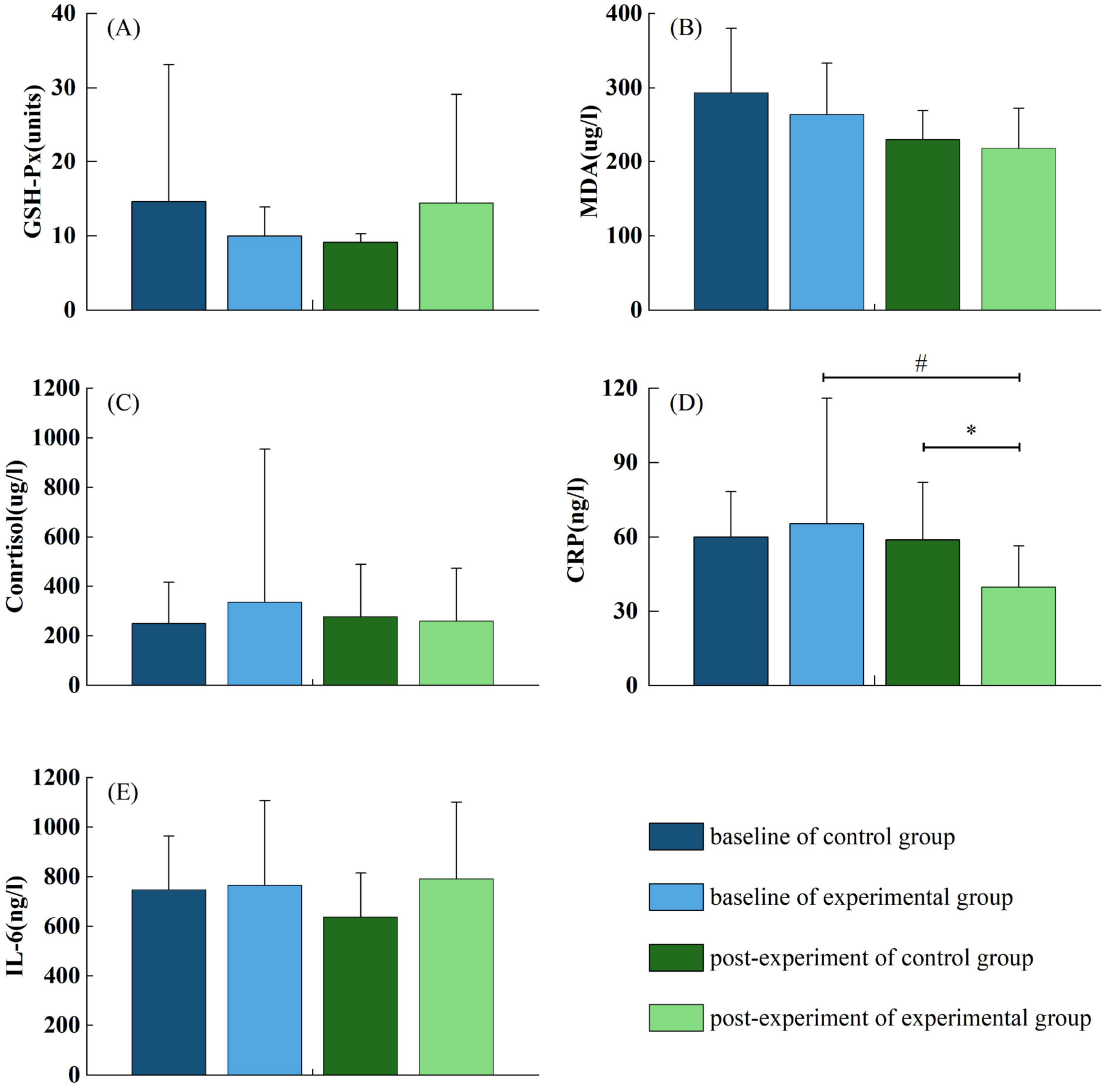
**(A–E)** Effects of forest bathing on hs-CRP, IL-6, Cortisol, MDA, and GSH-Px between the control and experimental group.

Among the measured biomakers, the level of hs-CRP was significantly lower in the experimental group than in the control group (39.86 ± 16.58 vs. 58.81 ± 23.18, *p* < 0.05), suggesting a reduction in systemic inflammation following exposure to the forest environment (Figure 6). However, no significant differences were observed between groups in other inflammatory and oxidative stress markers, including IL-6 (792.7 ± 309.22 vs. 639.09 ± 177.01, *p* > 0.05), cortisol (260.1 ± 213.23 vs. 277.58 ± 210.87, *p* > 0.05), MDA (218.47 ± 53.89 vs. 230.21 ± 38.9, *p* > 0.05), and GSH-Px (14.43 ± 14.66 vs. 9.14 ± 1.15, *p* > 0.05). These findings suggest that while forest bathing may exert anti-inflammatory effects, its influence on oxidative stress and hormonal regulation requires further investigation.

### 3.5 Effect of forest bathing on mood state

**Figure 7:**
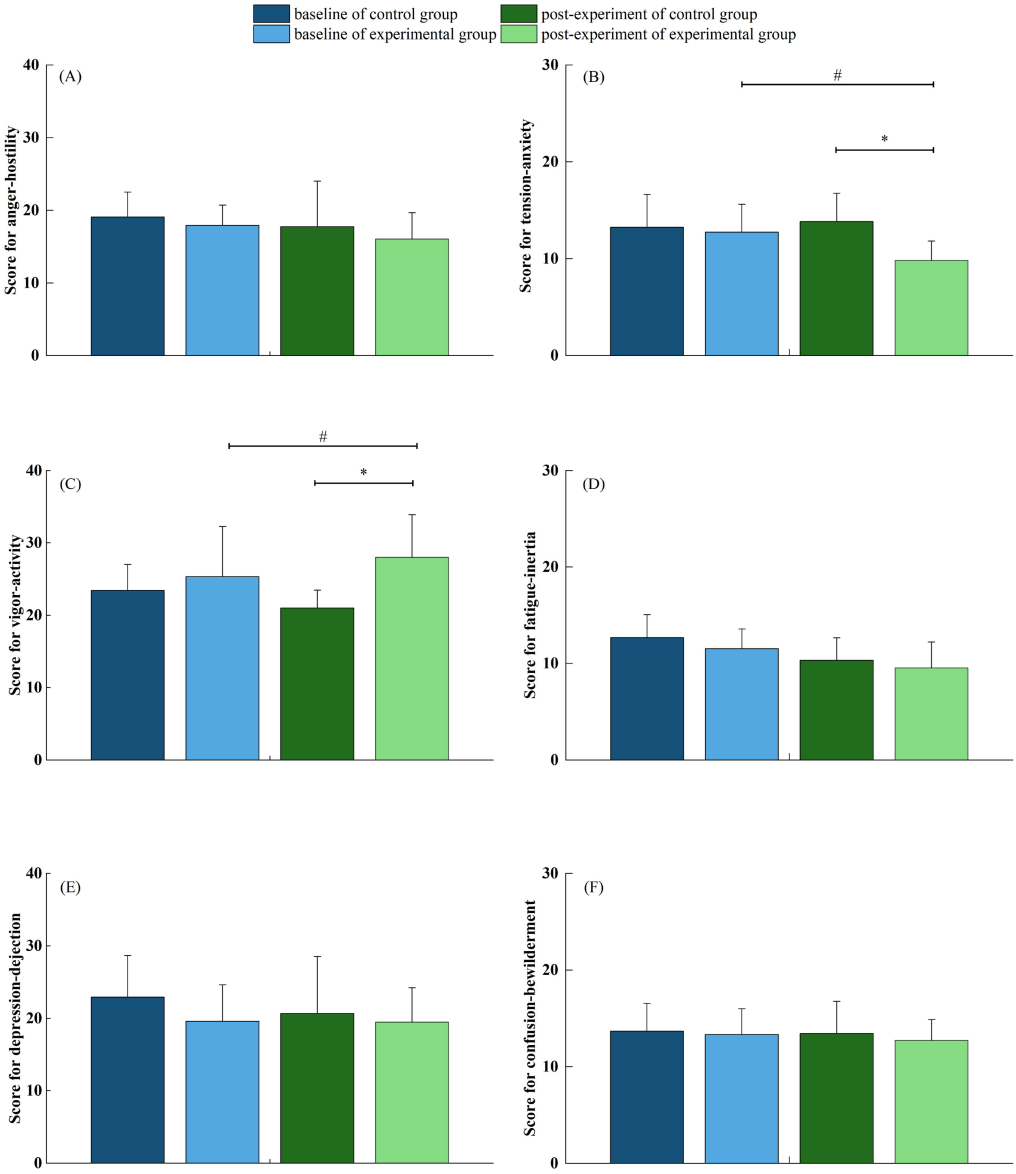
**(A–F)** Illustrate the differences in anger-hostility, tension-anxiety, vigor-activity, fatigue-inertia, depression-dejection, and confusion-bewilderment, respectively, between the experimental and control groups.

The assessment of mood states using the POMS questionnaire revealed notable differences between the experimental and control groups. The experimental group exhibited significantly lower scores in the tension-anxiety (T) subscale (9.83 ± 2.01 vs. 13.83 ± 2.92, *p* < 0.05), indicating reduced psychological stress, and significantly higher scores in the vigor-activity (V) subscale (28.0 ± 5.88 vs. 21.0 ± 2.45, *p* < 0.05), reflecting increased vitality and energy levels (Figure 7). While scores in the depression-dejection (D), anger-hostility (A), fatigue-inertia (F), and confusion-bewilderment (C) subscales were lower in the experimental group compared to the control group, these differences did not reach statistical significance (*p* > 0.05). Collectively, these results suggest that forest bathing may promote psychological well-being by alleviating negative emotional states and enhancing feelings of vitality in hypertensive older adult individuals.

## 4 Discussion

In this study, 36 older adult patients diagnosed with essential hypertension participated in a three-day experimental intervention. Participants were randomly assigned to either an experimental group (*n* = 24), which was exposed to the subtropical evergreen broad-leaved forest environment of Qianjiangyuan National Park, or a control group (*n* = 12) that remained in an urban setting. The objective was to evaluate the adjunctive therapeutic effects of forest bathing on essential hypertension. The therapeutic activities included in the experimental design were eight trigrams boxing, mountain walking, meditation, and tea ceremonies. Both groups adhered to a standardized schedule with identical commencement, conclusion, and duration for each activity, ensuring consistency across the interventions. Prior to the initiation of the experimental engagement, a statistical analysis revealed no substantial disparity among the physiological indicators of the subjects distributed across the two groups. However, upon conducting the same detect post-experimentation, it became evident that several of these physiological indicators exhibited statistically significant alterations.

### 4.1 Effect of forest bathing on BP

Our findings indicate that participants in the experimental group exhibited significantly lower SBP and DBP compared to the control group. One primary mechanism through which forest bathing promotes lower BP is the reduction of stress levels. Study suggests that forest-based interventions significantly reduce sympathetic nervous system activity, which is associated with stress, while enhancing parasympathetic activity, leading to a state of relaxation and subsequently lower blood pressure readings (24, 30, 31). Additionally, studies indicate that forest bathing can modulate stress hormones—such as cortisol—thereby contributing to cardiovascular health and the normalization of blood pressure (32, 33). Moreover, the effects observed in our study may also be attributed to the immune response enhancement noted in participants who engaged in forest therapy. Lyu et al. (34) found that forest bathing increased natural killer (NK) cell activity and related immune parameters, which can have cascading effects on stress and inflammation. This immune-response mechanism can indirectly affect cardiovascular health by reducing systemic inflammation, often correlated with elevated blood pressure levels. This aligns with our findings, which indicate lower hs-CRP levels in the experimental group, suggesting a possible link between immune modulation and blood pressure regulation.

### 4.2 Effect of forest bathing on HRV

HRV is widely recognized as a sensitive and non-invasive marker for autonomic nervous system (ANS) function and plays a critical role in cardiovascular regulation (35, 36). In the present study, exposure to the forest environment resulted in a significant increase in LF power and the LF/HF ratio in older adult patients with essential hypertension, compared to those exposed to an urban environment. This pattern indicates enhanced sympathetic nervous system modulation following forest bathing.

Although previous studies often reported that forest exposure promotes parasympathetic activation and reduces sympathetic drive, the results of our study suggest a different mechanism, particularly in older adult hypertensive individuals (37, 38). In this population, a moderate enhancement of sympathetic modulation may reflect a physiological adjustment aimed at improving cardiovascular adaptability and maintaining homeostasis. It is well documented that autonomic imbalance, characterized by either excessive sympathetic activity or impaired sympathetic responsiveness, contributes to poor blood pressure regulation and increased cardiovascular risk in hypertensive patients (39, 40). Therefore, a regulated increase in sympathetic activity, as indicated by the elevated LF power and LF/HF ratio, may represent a beneficial autonomic response rather than pathological overactivation.

The physiological mechanisms underlying this phenomenon are likely multifactorial. Forest environments, through their inherent restorative properties, may simultaneously reduce psychological stress and optimize autonomic responsiveness. Exposure to natural stimuli has been shown to decrease circulating stress hormones, including cortisol, adrenaline, and noradrenaline, thereby reducing allostatic load while promoting an adaptive cardiovascular response (1, 6, 37). Moreover, forest bathing may enhance baroreflex sensitivity, vascular tone regulation, and emotional resilience, all of which contribute to favorable shifts in HRV parameters.

In conclusion, our findings suggest that forest bathing modulates autonomic nervous system activity by facilitating a controlled increase in sympathetic nervous system modulation in older adult patients with essential hypertension. This autonomic adjustment may contribute to improved cardiovascular homeostasis, enhanced stress adaptability, and ultimately, reductions in systolic and diastolic blood pressure observed in the experimental group. Further studies are warranted to elucidate the long-term clinical implications of forest-based interventions in the management of hypertension and cardiovascular health.

### 4.3 Effect of forest bathing on inflammatory markers

Several biomarkers related to systemic inflammation and oxidative stress were investigated in this study, including hs-CRP, IL-6, cortisol, MDA, and GSH-Px (41, 42). Our results revealed a significant reduction in hs-CRP levels following forest bathing, indicating a decrease in systemic inflammatory burden in older adult patients with essential hypertension. hs-CRP is a sensitive marker of low-grade systemic inflammation, which is closely associated with cardiovascular risk and psychosocial stress pathways (43). Given that psychological stress is a well-established driver of chronic inflammatory processes, the observed reduction in hs-CRP may reflect the stress-relieving and homeostatic effects of the forest environment. Mechanistically, forest bathing may attenuate systemic inflammation through multiple pathways. Previous studies have demonstrated that exposure to natural environments reduces psychological stress and downregulates cortisol secretion, thereby restoring autonomic and endocrine balance (33, 44). Additionally, phytoncides—volatile organic compounds emitted by trees—have been reported to enhance natural killer (NK) cell activity and modulate immune responses, which could further contribute to the observed anti-inflammatory effects (45, 46).

In contrast, no significant changes were observed in other biomarkers, including IL-6, cortisol, MDA, and GSH-Px. Several possible explanations exist for these findings. One plausible reason is the relatively short duration of forest exposure in the present study, which may have been insufficient to induce detectable changes in these parameters. Previous investigations have suggested that longer-term or repeated exposure to natural environments is necessary to elicit substantial physiological improvements across multiple systems (2). Furthermore, certain biomarkers such as IL-6 and MDA may exhibit slower responsiveness to environmental interventions compared to more sensitive markers like hs-CRP (47).

It is also important to consider that the magnitude of physiological responses to forest bathing may depend on the cumulative “dose” of exposure, individual baseline health status, and underlying autonomic regulation. Thus, future studies incorporating longer intervention periods, multiple exposures, and longitudinal designs are warranted to comprehensively evaluate the effects of forest environments on inflammatory and oxidative stress pathways. In conclusion, the findings of the present study provide preliminary evidence that forest bathing may confer anti-inflammatory benefits in older adult hypertensive individuals, as indicated by reduced hs-CRP levels. However, its effects on other markers of oxidative stress and hormonal regulation remain inconclusive and merit further investigation.

### 4.4 Effect of forest bathing on mood state

Our study demonstrated significant improvements in mood among participants exposed to the forest environment, including reduced tension-anxiety and increased vigor-activity scores. These psychological improvements likely stem from physiological changes associated with reduced cortisol secretion, as previously discussed, contributing to relaxation and emotional well-being (8, 48). Additionally, nature exposure has been reported to stimulate parasympathetic nervous system activity, promoting a state of calm and emotional restoration essential for individuals experiencing chronic stress or anxiety (49, 50). As mentioned previously, improvements in cardiovascular parameters such as reduced blood pressure and enhanced HRV may also be associated with these positive mood changes (51).

Notably, the results showed that while the forest environment was effective in reducing negative mood states, the enhancement of positive mood states did not appear to be significant, as confirmed by the results of several studies (12, 52, 53). This suggests that while forest bathing can be effective in alleviating negative emotions, the enhancement of positive emotions may depend on a variety of factors, including length of exposure, individual characteristics, and environmental context. Future research may explore these moderators to optimize forest-based mental health interventions.

### 4.5 Prospects

This study provides empirical evidence supporting the therapeutic effects of subtropical evergreen broad-leaved forests on older adult patients with essential hypertension. However, several limitations should be acknowledged. Due to constraints related to funding, study duration, and participant selection criteria, this research was designed as a short-term intervention study rather than a long-term follow-up investigation. While our findings confirm the immediate physiological and psychological benefits of forest bathing, the long-term effects on cardiovascular regulation, autonomic nervous system adaptation, and chronic inflammation reduction remain unexplored (8). Future research should incorporate longitudinal studies to determine whether these acute improvements persist over extended periods and contribute to sustained health benefits. Additionally, individual variability in patient characteristics, including age, medication history, lifestyle factors, and baseline cardiovascular function, may influence the observed therapeutic outcomes. Expanding the sample size and diversifying the study population could enhance the generalizability of the findings (54).

The present study was conducted in a subtropical evergreen broad-leaved forest, a biome characterized by stable temperature and humidity, rich plant diversity, and elevated levels of negative air ions and biogenic volatile organic compounds. Such conditions may amplify the therapeutic potential of forest bathing by enhancing cardiovascular and mental health restoration (23). However, the geographic specificity of this forest type may raise questions about the ecological generalizability of the findings. Forests in boreal, temperate, or arid regions differ markedly in structure and microclimate, which may influence physiological and psychological responses. Future studies are needed to investigate how forest type, biodiversity, and climate interact with human health outcomes to refine nature-based intervention strategies across ecological zones.

Moreover, while this study primarily focuses on hypertension, forest bathing has also shown promising therapeutic effects for a range of other chronic conditions, including metabolic disorders such as diabetes, gastrointestinal dysfunction, and stress-related endocrine imbalances (5, 55, 56). These findings suggest that forest-based interventions may possess multifaceted health benefits, warranting broader exploration in diverse clinical contexts.

Moving forward, our research team aims to establish long-term monitoring programs to further investigate the health benefits of forest bathing. Future studies will focus on evaluating the cumulative effects of repeated forest bathing experiences, comparing different forest ecosystems, and integrating multi-disciplinary approaches to assess both physiological and psychological outcomes. Additionally, findings from this research may contribute to the development of evidence-based forest therapy programs, the implementation of public health strategies promoting nature-based interventions, and the establishment of forest wellness destinations for older adult populations. By advancing our understanding of the therapeutic potential of forest environments, we hope to provide scientific support for forest health policies and sustainable healthcare practices.

## 5 Conclusion

A three-day study demonstrated that a short-term forest bathing intervention in a subtropical broad-leaved evergreen forest was effective in lowering blood pressure, relieving systemic inflammation, improving autonomic regulation of cardiac function, and enhancing emotional well-being in older adult patients with essential hypertension. These findings provide strong empirical support for forest bathing as a valuable adjunctive therapy for the clinical management of older adult patients with essential hypertension. Further longitudinal studies are warranted to determine its sustained effects and to inform the wider implementation of forest therapy in preventive healthcare strategies.

## Data Availability

The original contributions presented in the study are included in the article/supplementary material, further inquiries can be directed to the corresponding authors.
